# Safety of Immersive Virtual Reality for the Management of Parkinson’s Disease

**DOI:** 10.3390/s24248188

**Published:** 2024-12-22

**Authors:** Daniela Pimenta Silva, Filipa Pona-Ferreira, Beatriz Santos, Pablo Campo-Prieto, Raquel Bouça-Machado, Joaquim J. Ferreira

**Affiliations:** 1 Centro de Estudos Egas Moniz, Faculdade de Medicina, Universidade de Lisboa, 1649-028 Lisbon, Portugal; dsilva7@edu.ulisboa.pt; 2 Laboratory of Clinical Pharmacology and Therapeutics, Faculdade de Medicina, Universidade de Lisboa, 1649-028 Lisbon, Portugal; 3 CNS—Campus Neurológico, 2560-280 Torres Vedras, Portugal; 4 HealthyFit Research Group, Galicia Sur Health Research Institute (IIS Galicia Sur), SERGAS-UVIGO, 36313 Vigo, Spain; pcampo@uvigo.gal

**Keywords:** neurorehabilitation, physiotherapy, exercise, virtual reality, virtual reality exposure therapy, Parkinson’s disease, safety, adverse events

## Abstract

Virtual reality (VR) has been used in research and clinical practice in the management of Parkinson’s disease (PD), potentially enhancing physiotherapy. Adverse events (AEs) associated with VR applications in PD have been poorly explored. We conducted a randomized controlled trial to compare two 12-week interventions using physiotherapy and immersive VR, and analyzed the frequency and type of AEs occurring in 30 people with PD. We reported 144 AEs (8.4% of the sessions), predominantly classified as mild and unrelated to the interventions. Two were serious AEs, one leading to study discontinuation. Notably, discomfort/pain, motor fluctuations, and falls were the most frequently reported, accounting for 63% of the total AEs. Five falls were definitely associated with the ‘sense of presence’ provided by the fully immersive VR system, which underscores the necessity for careful game selection when designing interventions for PD. Motor fluctuations may have been associated with various factors, which merit further investigation. We also explored the role of SSQ as a measure of cybersickness in PD. In conclusion, it is important to closely monitor and characterize AEs to ensure safety and efficacy in clinical practice as AEs may be more common than previously recognized in VR interventions in PD.

## 1. Introduction

Nonpharmacological interventions are one of the pillars of the complex management of neurodegenerative disorders. In Parkinson’s disease (PD), these interventions complement pharmacological and neurosurgical strategies and play a key role in targeting disease-specific impairments, tailored to each individuals’ needs, from mild to more advanced disease stages. These may include different combinations of physiotherapy and clinical exercise, occupational, speech, and psychology therapies, nutritional advice, social support, among others. Physiotherapy and clinical exercise are thus part of a holistic multidisciplinary care [1], guided by the European Physiotherapy Guideline for PD [2] and the principles of exercise by the American College of Sports Medicine (ACSM). Current recommendations are to combine various exercise modalities to engage people with PD in enjoyable and feasible regimens, while focusing on person-specific needs and individual preferences [2]. A recent Cochrane review confirmed that most types of physical exercise are beneficial in improving motor signs and quality of life of individuals with PD [3].

Notwithstanding the proven efficacy of physiotherapy, safety is one important but often neglected subject [3,4]. The 2023 Cochrane review demonstrated that only 85 out of the 156 included randomized controlled trials (RCT) that provided some kind of safety data, while only 28 studies reported the occurrence of adverse events (AEs) [3]. As people with PD tend to underreport adverse drug reactions spontaneously [5], it is difficult to determine whether the low frequency of AEs in studies on nonpharmacological interventions follows an actual absence of an AE or an inadequate assessment. Using a structured and systematic approach to record AEs during and after specialized physiotherapy sessions for Parkinsonian patients, our study group previously reported a total of 128 AEs, which occurred in 7% of the sessions [6]. Although physiotherapy should still be considered safe, this study underlines the need to actively inquire patients about adverse reactions to exercise since they may be more frequent than usually reported [6].

Virtual reality (VR) and exergaming are being increasingly used in research studies and clinical practice as tools to complement traditional physiotherapy and exercise. By providing multisensory stimuli and demanding constant interactivity, both VR and exergaming enhance feedback about performance and results, challenge motor and cognitive processes simultaneously, and motivate individuals to actively engage in exercise. Several studies have demonstrated the efficacy of VR in several clinical outcomes [7,8,9,10,11,12]. Nevertheless, as in interventions with traditional physiotherapy, AEs have been poorly reported. Cybersickness is a common concern, especially in studies using head-mounted displays (HMDs) [13], which are technologies promoting immersive experiences by cancelling the real-world surroundings thus increasing the sense of presence within the virtual environment [14]. Yet, it appears to be an uncommon adverse reaction, even when assessed through questionnaires [15,16], while other AEs associated with exercise might be overlooked (e.g., falls, pain) [6].

In this paper, we aim to explore the occurrence of AEs during interventions combining immersive VR (IVR) systems with physiotherapy in the management of PD. We will further characterize the type of AEs and their relationship with the interventions. We hope to guide future experimental studies using VR to improve AEs recognition and ensure safety and efficacy.

## 2. Methods

We conducted an open label rater-blinded, randomized controlled trial at CNS-Campus Neurológico, a specialized movement disorder center in Portugal, to evaluate the efficacy, safety, and usability of using IVR through an HMD device as a complement to a specialized physiotherapy program into the management of functional mobility with cognitive dual-tasking in PD. The study protocol is further described in ClinicalTrials.gov under the identifier NCT06052930. For the purpose of this paper, we will focus on AE-related data to characterize the frequency, type, and severity of AEs and their causal relationship with interventions that combine physiotherapy and IVR. We will also explore cybersickness-related symptoms.

Individuals with mild to moderate PD (Hoehn and Yahr (HY) stage 1 to 3), and able to perform Timed Up and Go (TUG) test unassisted in less than 11.5 s [17,18] on medication were randomly assigned to either perform a 12-week combination program with PD-specialized physiotherapy plus IVR (VR group, VRG), or a sequential program starting with 6 weeks of PD-specialized physiotherapy alone followed by 6 weeks of the combination of physiotherapy and VR (sequential group, SG).

Each IVR training session started with 6 to 8 min of warm-up, followed by 20 min of boxing, using a commercially available HMD, the HTC Vive™ Pro (HTC Corp., Taoyuan City, Taiwan). The NVIDIA^®^ VR Fun House experience is available on the Steam VR store (https://store.steampowered.com/, accessed on 15 October 2024). It was used as a warm-up, where participants were required to perform different tasks at a funfair, such as throwing objects, archery, or handling a sword. The following boxing experience was a high-intensity training performed using the exergame BOX VR (available on the Steam Store https://store.steampowered.com/, accessed on 15 October 2024). It involved performing boxing movements in response to the randomly presented stimuli, which required coordinated movements of the upper limbs, trunk, head, and lower limbs. It incorporated variations in stance, squats, and lateral movements within a gym scenario boosted by motivating music. Regarding the PD-specialized physiotherapy sessions, it was delivered in accordance with recommendations from the European Physiotherapy Guideline for PD [2], lasting 60 min each session. IVR and physiotherapy were delivered on the same day, three times per week. Participants were supervised by the study investigators who were physiotherapists experienced in PD rehabilitation (FPF and BS), and a neurologist experienced in IVR training in individuals with PD (DPS).

All AEs were systematically recorded by the investigators using a similar approach to a previously applied approach for a physiotherapy intervention in Parkinsonian patients [6]. Investigators were asked to remain vigilant and to document any type of event. A list of AEs was provided in advance to enhance their identification and ensure consistency in description, regardless of whether the AE was observed during sessions or reported spontaneously by participants. Therefore, we used an adapted form where investigators would describe the event, detailing the type of AE, timing (whether it occurred during or after the session), the intervention associated with it, severity, and investigators’ assessment of its causality. For the purpose of this study, AEs were defined as any unfavorable and unintended sign, symptom, or disease temporally associated with the physiotherapy and/or IVR sessions that may or may not be considered related to the intervention [6,19]. Severity was graded according to the investigators’ best clinical judgment using the Common Terminology Criteria for Adverse Events (CTCAE) as mild, moderate, severe, life-threatening, or death-related [6,20]. A serious AE was considered when the participant outcome was death, life-threatening, hospitalization, disability, or permanent damage, or an intervention was required to prevent harm [21]. Regarding causality, investigators categorized each AE as definitely, possibly, improbably, or unrelated to the interventions, using the World Health Organization Upsala Monitoring Centre (WHO-UMC) causality assessment system [6,22].

In addition to this detailed approach of AE reporting, we used the Simulator Sickness Questionnaire (SSQ) [23,24] to actively enquire participants about cybersickness-related symptoms at baseline, at the end of each session during the first two weeks, and at the end of the 6th and 12th weeks, thus enabling us to evaluate the evolution of SSQ scores over time, as previously suggested [25]. The SSQ was originally designed to measure simulator sickness associated with military flight simulators, and consists of 16 items assessed on a four-point scale, according to the degree of presence of the symptom (0—absent; 4—severe). This tool has been widely used to measure the perception of cybersickness symptoms related to VR systems in the general population [23].

We characterized the sample at baseline and the AEs using a descriptive analysis.

## 3. Results

Thirty participants were randomly assigned to one of the training groups. The VRG had a median [interquartile range, IQR] age of 61 [58, 66] years, a median disease duration of 7 [6, 19] years, a median MDS-UPDRS part 3 score of 31 [23, 35.5], and a median Hoehn and Yahr (HY) of 2 [2, 2]. The SG had a median age of 68 [62, 72.5] years, a median disease duration of 9 [5, 11.5] years, a median MDS-UPDRS part 3 score of 23 [13, 24.5], and a median HY of 2 [2, 2]. Baseline characteristics are summarized in Table 1.

The total number of sessions held was 1717. The VRG attended 1012 sessions, including both VR and physiotherapy sessions during the whole study duration, whereas the SG attended 705 sessions, comprising physiotherapy alone during the first 6 weeks and physiotherapy plus IVR during the following 6 weeks of this study.

Table 2 presents the AEs that occurred in each group throughout this study. In total, 144 AEs were reported over the 12-week intervention, which corresponds to 8.4% of the attended sessions. Eighty-six AEs occurred in the VRG, accounting for 8.5% of the attended sessions, while 58 were recorded in the SG, accounting for 8.2% of the attended sessions. A total of 23 (76.7%) participants experienced more than one AE, with 12 from the VR group and 11 from the sequential group. For one patient in the sequential group, the AE was reason to discontinue the intervention shortly after it started.

### 3.1. Frequency and Type of Adverse Event, Setting, and Causality

The most frequent AEs were discomfort/pain, motor fluctuations, and falls, which occurred in 2.7%, 2%, and 0.6% of total attended sessions, respectively. These three events represented 63.2% of all AEs. Overall, 77 (53.5%) AEs were recorded during or after IVR sessions, while physiotherapy sessions were associated with 30 (20.8%) events.

Most AEs (52.1%) were classified as unrelated or improbably related to study interventions. For the IVR training, 28 AEs were considered possibly related, and 16 definitely related, while for the physiotherapy sessions, 14 were deemed possibly related, and 2 definitely related. The AEs possibly to definitely related to IVR training included motor fluctuations (*n* = 17), discomfort/pain (*n* = 16), falls (*n* = 5), fatigue/tiredness (*n* = 3), irritability/frustration (*n* = 2), and dizziness (*n* = 1). The AEs potentially related to physiotherapy sessions consisted of discomfort/pain (*n* = 6), hypotension (*n* = 3), hyperhidrosis (*n* = 3), cramps (*n* = 1), dizziness (*n* = 1), fatigue/tiredness (*n* = 1), and hematuria (*n* = 1). Fifteen (10.4%) AEs were experienced during or after both the IVR and physiotherapy sessions.

In the following sections, we will further characterize the three most common AEs.

#### 3.1.1. Discomfort/Pain

Experienced by 15 participants, discomfort/pain occurred during or after sessions in 30 out of the 46 events, more frequently during IVR training (*n* = 22). The nature of the discomfort/pain was presumably musculoskeletal in most cases, namely lumbar pain and arthralgia. However, in some cases (*n* = 12), the pain was located on the side most affected by PD.

#### 3.1.2. Motor Fluctuations

A diary of motor complications had not been planned; therefore, the reported motor fluctuations were those observed by the investigators during sessions.

The 34 events were noticed in five participants, occurring more frequently during IVR training sessions (*n* = 28), typically after 10 to 18 min of intense exercise with the IVR boxing game. In some cases, motor fluctuations had an impact on exercise performance, with sessions having to be interrupted to rest, or decrease the difficulty of the game.

Twenty-six of the total events were due to two participants only. In both cases, participants already presented motor fluctuations at baseline, as measured by item 3 of the MDS-UPDRS part 4. Yet, they judged these events potentially triggered by the physical exertion and fatigue, as they occurred suddenly and unexpectedly in the middle of the sessions. Therefore, the exercise may have triggered a ”wearing-off” phenomenon or exacerbated an OFF episode, since the motor fluctuations appeared approximately 3.5 h after the previous dose intake. These were similar to those occurring at home, comprising a reemergence of Parkinsonian tremor, bradykinesia, rigidity, painful OFF dystonia, gait disturbance, and freezing of gait, as described by participants.

#### 3.1.3. Falls

**Figure 1:**
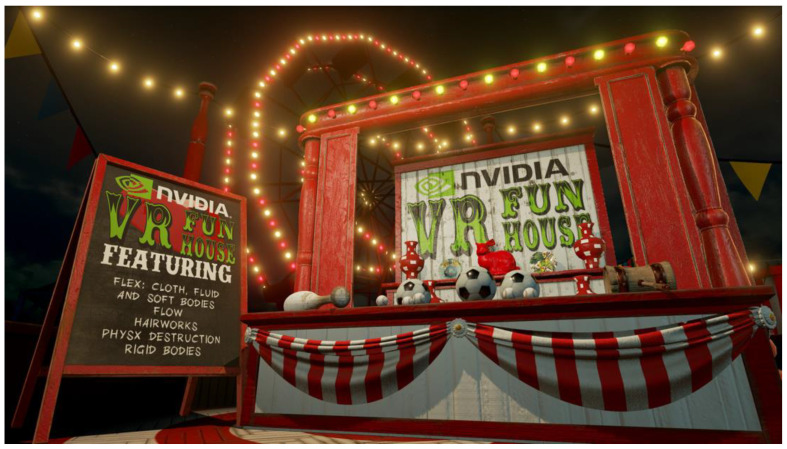
Warm-up funfair scenario. Available at the Steam VR platform.

Eleven falls were reported during this study, either self-reported by participants or observed during sessions. Five of these uninjured falls occurred in exactly the same circumstance during the IVR training sessions, which were deemed definitely related to the intervention, as a consequence of the ‘sense of presence’ provided by the HMD. In the funfair scenario, participants were required to grab balls and other objects from a virtual table, and throw them to break dishes displayed on a shelf. As represented in Figure 1, the balls had different sizes and were distributed on the table, with some being more reachable than others. These five falls were caused by participants leaning over the virtual table with the support of one hand to grab a ball further away, thus reaching a point of imbalance in the real world. These participants were HY of 2, and did not have significant postural instability. All the other falls occurred after or between sessions and were considered not related to the study trainings.

### 3.2. Severity of Adverse Events

AEs were mostly mild (*n* = 99, 68.8%), representing mild symptoms or asymptomatic. Forty-one (28.5%) were graded as moderate, with minimal, local, or noninvasive intervention indicated, while two (1.4%) were considered severe or medically significant, limiting self-care activities of daily living. Two AEs (1.4%) were classified as serious, requiring inpatient hospitalization and urgent surgical treatment. One involved a fall at home resulting in a hip fracture, and the other an incarcerated hernia. Both severe and serious AEs were deemed unrelated to the interventions.

### 3.3. Cybersickness

**Figure 2:**
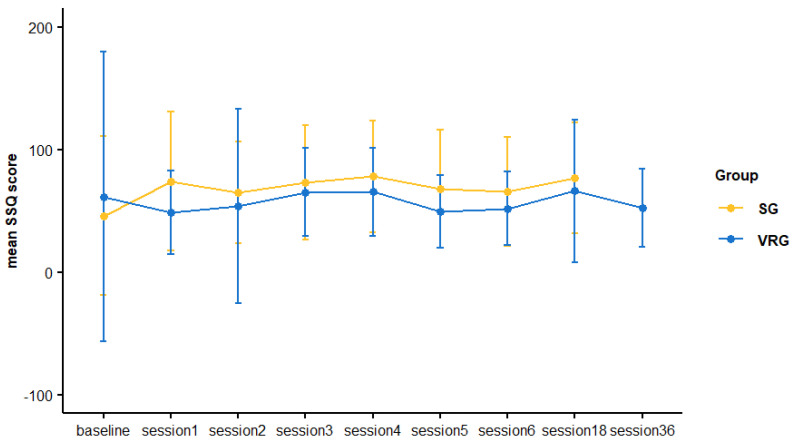
Evolution of the Simulator Sickness Questionnaire (SSQ) scores over time by study group. VRG—virtual reality group; SG—sequential group.

The baseline mean SSQ score was 54.2 (±95.56), already indicating concerning levels of cybersickness symptoms [23] even before using the IVR system, which may simply represent the overlap between the SSQ 16 items and PD symptoms. Nevertheless, as represented in Figure 2, the mean SSQ scores remained stable throughout this study relative to baseline, varying from 52.2 (±31.86) and 73.3 (±42.05). The SSQ items more frequently scored above 0 were fatigue and sweating, with some participants mentioning increased salivation, blurred vision, eyestrain, difficulty focusing and concentrating, fullness of head, and headache, some of which were already present at baseline.

## 4. Discussion

In this paper, we comprehensively described the AEs reported during an RCT investigating the combination of physiotherapy and IVR in the management of PD. Using a structured approach, we found that 144 AEs were recorded over 12 weeks, corresponding to 8.4% of the sessions held. This rate is lower than typically reported for pharmacological interventions, comparable to the AE frequency observed in physiotherapy sessions for Parkinsonian patients [6], and higher than usually reported for VR interventions [16,26].

To the best of our knowledge, this is the first time that such a detailed description has been made to characterize AEs during interventions using VR in PD. While we recognize that the AE profile may change between different types of VR, many studies lack information regarding the occurrence of AEs [12,26,27,28,29,30,31,32,33,34]. Other studies report no AE [15,35,36,37,38,39,40] which, despite reassuring, raises questions about whether AEs have been adequately monitored or reported in these clinical trials. Studies reporting AEs infrequently attribute them to any of the interventions, or there are insufficient details about causality. Two well-conducted RCTs, the V-TIME study [7] and the Park-in-Shape trial [8], have reported AEs in aerobic training using VR. The V-TIME study, comparing treadmill training alone with treadmill training augmented by VR in elderly people with a risk of falls, reported 28 AEs over 6 weeks, all leading to study discontinuation, five of which were serious AEs [7]. Although the approach is not fully described, the authors state that the causality was investigated and none were related to the interventions [7]. Regarding the Park-in-Shape trial, 27 AEs were reported in individuals with PD performing a home-based training on a stationary bicycle enhanced by VR during 6 months; three were serious AEs [8]. The authors reported that seven AEs were potentially exercise-related, including arthralgia, back pain, and palpitations [8]. Nevertheless, there is no detailed description of how AEs were documented and characterized.

In our study, three AEs accounted for more than half (63%) of the total events, including discomfort/pain, motor fluctuations and falls. Pain and falls were already described as being among the most frequent AEs in physiotherapy interventions and exercise-related injuries [6,41,42,43]. Regarding pain, it has been considered related to endurance exercises in 8.5% to 23.9% of the patients, especially when exercises are strenuous [8,41]. We found that discomfort/pain were probably/definitely related to the interventions in 43% of the participants, mostly to IVR training, which was also aerobic and intense. Although being the most frequent AE, it occurred in less than 3% of the attended sessions and was mostly mild in severity. Similar to other studies, pain was presumably musculoskeletal in most cases, including back pain and arthralgias. However, in 12 cases, the pain occurred in the most affected side of the disease. While we could not establish the exact cause or associate it with OFF periods, we speculate that it may be an exacerbation of a previous pain, caused by the fast and big amplitudes that the VR boxing training demanded, which challenged the spontaneously slower and smaller movements of the more rigid and bradykinetic limb. In addition, hand dominance and the disease asymmetry may have added to this limiting factor. To our knowledge, this phenomenon has not been previously described in VR users with PD, and further studies should investigate its nature and impact, to enable the development of an individualized approach for susceptible individuals.

Falls in physiotherapy and exercise interventions, with or without VR, have been rare and non-injurious, albeit circumstances being often poorly described [7,8,41,42,43]. Previous studies using fully immersive VR systems in older adults and in people with PD have no reported falls, thus considering them safe for boxing training [40] and walking [15], including in an antigravity treadmill setting [16], although patients were secured with safety harnesses to prevent falls. In our study, we encountered 11 falls, 6 of them unrelated to the intervention, with one resulting in a hip fracture. Interestingly, we observed five falls occurring under identical conditions, which could not be better explained by underlying postural instability. Instead, these falls were definitely caused by the ‘sense of presence’ that the fully immersive virtual environment induced [44]. In our view, the discrepancy between the virtual environment and the real-word actions, along with the HMD’s ability to cancel the real surroundings, makes IVR systems susceptible to these types of events. Therefore, we suggest to avoid games that place users in imbalanced positions or involve actions incongruent with the physical environment, and to carefully provide safety measures to prevent serious complications. In addition, it is advisable to supervise fully immersive VR interventions at all times, or provide exergames with sitting positions to prevent falls in home-based use [39]. People with an increased risk of falls and postural instability may present additional limitations to the implementation of IVR, thus its safety in this group of individuals needs careful investigation.

Motor fluctuations have not been described in other studies with VR, while we observed them in 2% of the attended sessions. Three main reasons may have contributed to the increased rate of this AE in our study. First, considering that participants already experienced motor fluctuations at baseline and that they were observed approximately 3.5 h from the last levodopa intake, these episodes likely reflected an exacerbation of their usual “wearing-off” phenomenon caused by exercise. Second, the same patient may have presented the same AE several times due to the episodic nature of motor fluctuations and their possible relation to the moment of exercise. Third, the investigators’ had an increased awareness to report all events during the study period. Although sessions were scheduled to be optimized by participants’ ON state based on their preferred timing and perceived ideal exercise window, these events often emerged suddenly after some time of intense aerobic exercise. We hypothesize that physical exertion may have either worsened motor fluctuations or triggered OFF episodes. However, it is controversial whether physical exercise has a triggering or an attenuating effect on OFF symptoms [45,46]. For some patients, exercise is a coping strategy to overcome an OFF episode [45], whereas for others it can be a source of increased fatigue [47], which is a trigger of OFF episodes [45]. A recent study found that people with advanced PD (PD with motor complications, mean disease duration of 8.7 ± 5.9 years) were more likely to worsen motor scores immediately after physical exercise than patients with milder disease, which did not seem to be caused by changes in the levodopa pharmacokinetics after exercise [46]. While it may be an explanation for this ‘acute’ phenomenon in our study, we can not exclude that other factors unrelated to exercise may have played a role, such as stress, anxiety, or lack of sleep [45]. In addition, it is not known whether the type of VR system may have a triggering effect on motor fluctuations, particularly regarding the intensity, velocity, and variety of visual stimuli, the complexity of the VR environment, and the level of immersion.

Cybersickness is a frequent safety concern in studies using IVR, though in practice it is rarely reported. While SSQ was originally designed for military flight simulators, it has been used to measure motion sickness related to the use of VR systems [25]. These symptoms are then clustered into categories representing symptoms of nausea, oculomotor disturbance, and disorientation, and summed up into a total simulator sickness score [23]. Based on the original military sample, total scores can be associated with negligible (under 5), minimal (between 5 and 10), significant (between 10 and 15), and concerning (between 15 and 20) symptoms [23]. Although the scale seems appropriate for VR research, measuring cybersickness only after exposure presumes that participants are perfectly well before the exposure [25]. In fact, comparing the SSQ 16 items and PD symptoms, the overlap is unquestionable (e.g., general discomfort, fatigue, difficulty focusing, increased salivation, sweating, nausea, difficulty concentrating, and dizziness), thus explaining why mean scores already indicated concerning levels of cybersickness before VR exposure in our participants. Therefore, while SSQ might not be totally appropriate in individuals with PD, we measured SSQ at baseline and at different timepoints, following prior recommendations [25], and we found that mean scores remained stable across all assessments, including when compared to baseline. The most common scored symptoms after VR exposure were fatigue and sweating, which are most likely related to the boxing aerobic exercise. Our findings are consistent with previous studies suggesting that motion sickness may not be a significant concern in most cases [40,48]. Nonetheless, factors such as game content, type of visual stimulation, time of exposure, and age, which have been linked to VR-related sickness [13], should be taken into consideration when applying IVR to PD patients.

Our study has some limitations. First, we included participants with mild to moderate PD, with and without motor complications, which may have different AE profiles. Second, session schedules were chosen according to participants’ preferences and their reported optimal ON window, which in some cases may not necessarily mean the best time to perform intense physical activity, especially in individuals with known motor fluctuations not used to exercising. This may have influenced the increased reporting of OFF episodes and other AEs during sessions. Scheduling exercise between one to two hours from the last levodopa intake may better overcome this limitation. Third, we did not perform a comparative analysis between study groups nor between VR and physiotherapy interventions, therefore, we cannot conclude on the causality of the increased rate of AEs. Despite these limitations, we believe our study provides critical insights for informing future research in AE reporting in interventions for PD.

Taken together, we consider that physiotherapy combined with IVR can be considered safe for the management of PD, with AEs occurring in fewer than 10% of sessions, being mostly mild and not related to the interventions themselves. The frequency and type of AEs were similar to those previously described for traditional physiotherapy; however, we highlight the overall higher rate when compared to what was previously reported for VR interventions, as well as the frequent occurrence of motor fluctuations. This difference likely reflects the investigators’ increased awareness for reporting, alongside our structured approach to identifying and characterizing these events. Further investigation is needed to ascertain whether the type of VR, the level of immersion (immersive vs. non-immersive), visual stimulus intensity, session duration, the exercise intensity, disease duration, and timing of the last levodopa intake may influence the AEs profile during VR interventions in PD. Falls are a significant concern when using IVR systems, especially in people with PD, and should be anticipated when selecting games or devices.

## 5. Conclusions

We suggest that AEs may be more common in sessions combining VR and physiotherapy than previously recognized, although we still consider it a safe intervention. It is therefore important to plan ahead with risk minimization strategies and ensure safety and efficacy when using VR for the management of PD. Future studies should replicate our approach in AE reporting to better characterize the relationship between the events and type of VR intervention.

## Figures and Tables

**Table 1:** Baseline participants’ characteristics.

	VR Group (*n* = 15)	Sequential Group (*n* = 15)
Age (years), median [IQR]	61 [58, 66]	68 [62, 72.5]
Gender (men), n (%)	9 (60)	8 (53.3)
Education, n (%)		
Low	5 (33.3)	7 (46.7)
Medium	4 (26.7)	1 (0.07)
High	6 (40)	7 (46.7)
Disease duration (years), median [IQR]	7 [6, 10]	9 [5, 11.5]
First symptom, n (%)		
Tremor	7 (46.7)	7 (46.7)
Bradykinesia-rigidity	7 (46.7)	7 (46.7)
Other	1 (0.07)	1 (0.07)
MDS-UPDRS part 3, median [IQR]	31 [23, 35.5]	23 [13, 24.5]
HY, median [IQR]	2 [2, 2]	2 [2, 2]

IQR—interquartile range (25th percentile; 75th percentile); MDS-UPDRS—Movement Disorder Society Unified Parkinson’s Disease Rating Scale; HY—Hoehn and Yahr.

**Table 2:** Reported adverse events.

Adverse Event (AE)	N (%)	VR Group		Sequential Group	
		0–6 Week	6–12 Week	0–6 Week	6–12 Week
Discomfort/pain	46 (2.7)	15 (2.9)	6 (1.2)	5 (2.0)	20 (4.3)
Motor fluctuations	34 (2.0)	16 (3.1)	14 (2.8)	1 (0.4)	3 (0.7)
Falls	11 (0.6)	3 (0.6)	0	2 (0.8)	6 (1.3)
Fatigue	9 (0.5)	5 (1)	0	1 (0.4)	3 (0.7)
Near falls	8 (0.5)	4 (0.8)	2 (0.4)	0	2 (0.4)
Hypotension	6 (0.3)	1 (0.2)	0	4 (1.6)	1 (0.2)
Dizziness	3 (0.2)	0	1 (0.2)	0	2 (0.4)
Hyperhidrosis	3 (0.2)	3 (0.6)	0	0	0
Dyskinesias	3 (0.2)	3 (0.6)	0	0	0
Irritability/frustration	2 (0.1)	0	1 (0.2)	0	1 (0.2)
Gut disturbance	2 (0.1)	0	1 (0.2)	0	1 (0.2)
Headache	2 (0.1)	1 (0.2)	0	0	1 (0.2)
Impulse control disorder	2 (0.1)	0	2 (0.4)	0	0
Muscle soreness	2 (0.1)	2 (0.4)	0	0	0
Hypertension	1 (0.06)	1 (0.2)	0	0	0
Numbness/tingling	1 (0.06)	1 (0.2)	0	0	0
Syncope	1 (0.06)	1 (0.2)	0	0	0
Sleep disturbance	1 (0.06)	0	1 (0.2)	0	0
Dry mouth	1 (0.06)	0	0	1 (0.4)	0
Hematuria	1 (0.06)	0	0	1 (0.4)	0
Cramps	1 (0.06)	0	0	0	1 (0.2)
Gout	1 (0.06)	0	0	0	1 (0.2)
Chin rash	1 (0.06)	0	0	0	1 (0.2)
Complicated hernia	1 (0.06)	0	1 (0.2)	0	0
Flu-like symptoms	1 (0.06)	1 (0.2)	0	0	0
Total	144 (8.4)	57 (11.2)	29 (5.8)	15 (6.1)	43 (9.3)

Values are presented as the total count of occurrences throughout this study and as the percentage relative to the total number of attended sessions, per group. Attendance was as follows: total attended sessions = 1717; sessions attended by the VR group = 1012 (0–6 week = 510 sessions, 6–12 week = 502 sessions); and by the sequential group = 705 (0–6 week = 245 sessions, 6–12 week = 460 sessions).

## Data Availability

The original contributions presented in this study are included in the article. Further inquiries can be directed to the corresponding author.
